# The Role of Bacterial Colonization of the Suture Thread in Early Identification and Targeted Antibiotic Treatment of Surgical Site Infections: A Prospective Cohort Study

**DOI:** 10.3390/ijerph17124416

**Published:** 2020-06-19

**Authors:** Francesco Iovino, Federica Calò, Consiglia Orabona, Alessandra Pizza, Francesca Fisone, Pina Caputo, Alessandra Fusco, Margherita Macera, Nicola Coppola

**Affiliations:** 1 Department of Translational Medical Sciences-Division of General Surgery, University of Campania Luigi Vanvitelli, 80131 Naples, Italy; francesco.iovino@unicampania.it (F.I.); orabonaconsiglia@hotmail.it (C.O.); alessandra.pizza1980@libero.it (A.P.); fisone.francesca@gmail.com (F.F.); 2 Department of Mental Health and Public Medicine—Infectious Diseases Unit, University of Campania Luigi Vanvitelli, 80131 Naples, Italy; fede.calo85@gmail.com (F.C.); macera.margherita@libero.it (M.M.); 3 Department of Experimental Medicine, Section of Microbiology and Clinical Microbiology, University of Campania “Luigi Vanvitelli”, 80131 Naples, Italy; pinacaputo@hotmail.it (P.C.); Alessandra.FUSCO@unicampania.it (A.F.)

**Keywords:** surgical site infection, antibiotic prophylaxis, bacterial colonization

## Abstract

*Background:* The aim of the present study is to investigate the role of the colonization of suture thread to identify patients at risk of developing a surgical site infection (SSI) after clean surgical procedures. *Methods:* Patients who underwent elective clean surgery procedures at the Surgery Unit of the AOU-University of Campania Luigi Vanvitelli in a 21-month period were prospectively enrolled. For each patient, a synthetic absorbable thread in Lactomer 9-1 was inserted into the surgical site at the end of surgery and microbiologically evaluated after 48 h. Antibiotic prophylaxis was chosen according to international guidelines. *Results:* A total of 238 patients were enrolled; 208 (87.4%) of them were subjected to clean procedures without the placement of prosthesis, and 30 (12.6%) with prosthesis. Of the 238 patients, 117 (49.2%) underwent an antimicrobial prophylaxis. Overall, 79 (33.2%) patients showed a bacterial colonization of the thread: among the 208 without the implantation of prosthesis, 19 (21.8%) of the 87 with antibiotic prophylaxis and in 58 (47.9%) of the 121 without it; among the 30 patients with the implantation of prosthesis, only two patients showed a colonized thread. The patients with antibiotic prophylaxis developed a colonization of the thread less frequently than those without it (17.9% vs. 47.9%, *p* < 0.001). SSI was observed in six (2.5%) patients, all of them showing a colonized thread (7.6% vs. 0%, *p* < 0.001). The bacteria identified in colonized threads were the same as those found in SSIs. *Conclusions:* Our study presents a new method that is able to precociously assess patients who have undergone clean procedures who may develop SSI, and identify the microorganism involved.

## 1. Introduction

Surgical site infections (SSIs) are among the most common healthcare-associated infections, and are associated with longer post-operative hospital stays, intensive care admission and higher mortality [1].

The burden of SSIs in the United States is estimated at about 2% of all patients who undergo an operation [2,3], while in European countries the percentage of SSIs vary between 0.5% and 10.1%, depending on the type of surgical procedure [1]. In fact, some types of operations, such as colonic surgery, report much higher rates of infection [4]. The latter also significantly varies in relation to the presence or absence of prosthetic material. Indeed, in 2017 the infection rates reported in our region, Campania, southern Italy, ranged between 1.5% and 1.9%, respectively, in procedures with or without the implantation of prosthesis [5].

Most surgical site infections are superficial and occur near or at the incision site; however, some may be deeper, involving underlying tissue spaces and organs within 30 days of the surgical procedure (or up to 90 days for implanted prosthetics) [6].

The rates of SSIs vary depending on the type of surgery and on the different degree of contamination. Risk factors for SSIs include age, type and length of operation, surgery complexity, American Society of Anesthesiologists’ (ASA) classification, obesity/underweight, tobacco or steroid use, preoperative sepsis, perioperative blood transfusion, hyperglycemia, comorbidities such as diabetes mellitus, renal failure, cancer and chronic obstructive pulmonary disease, surgical wound classification, and postoperative drainage [2,7,8].

SSIs are largely preventable. Antibiotic prophylaxis aims to reduce the bacterial contamination of the surgical site and, therefore, the risk of infection. Procedural prophylaxis (before or during surgery) is not indicated for all surgeries, especially minor procedures or most clean procedures without the implantation of prosthesis [9].

In many SSIs, the responsible pathogens originate from the patient’s endogenous flora. Although the distribution of the microorganisms responsible for SSIs varied by type of surgical procedure, for all types of surgical procedure, Gram-positive cocci were the most frequently reported microorganisms [1].

Any suspected SSI should have wound swabs taken for culture at the wound site, especially if a purulent discharge is present. However, it is necessary to carry out the skin swab correctly (avoiding the edges of the wound) in order to reduce, where it is possible, skin flora contamination.

Concerning SSI management, clinicians need to promptly recognize it to carry out a tailored management according to the specific condition of the patient and to the local rates of antibiotic resistance, and have to start an empirical antibiotic therapy, then adapting it to the results of the culture. In a previous study [10], we analysed the role of a suture thread in patients who underwent all types of surgical procedures, in order to identify the patients who would develop a surgical site infection, showing a sensitivity of this method for SSI of 100% and a specificity of 68.7%

The aim of this study was to investigate the role of this method, which exploits the colonization of a suture thread to precociously identify the at-risk patients who have undergone only clean surgical procedures.

## 2. Methods

### 2.1. Study Design and Patient Selection

This study was a prospective monocentric national cohort study, including consecutive patients undergoing clean elective surgery at the Surgery Unit of the AOU-University of Campania Luigi Vanvitelli, Naples, Italy in a 21-month period (from July 2017 to March 2019).

For this analysis, only adult (age ≥ 18 years) patients subjected to a clean surgical procedure were included.

The study was approved by the Ethics Committee of the teaching hospital of Campania Luigi Vanvitelli (no. 960/2015). All procedures were carried out in accordance with international guidelines and with the Helsinki Declaration of 1975, revised in 1983. All patients enrolled in the study signed an informed consent for surgery, the collection of intra-operative samples (including the suture thread), and the anonymous use of their data for research purposes.

### 2.2. Variables and Definitions

Data collected from all patients enrolled included: demographics; the presence of underlying chronic disease in accordance to the ASA score criteria; and information on each surgical procedure. Data regarding the duration of hospitalization and the average duration of the surgical procedure were also collected.

The preoperative patient assessment included a complete clinical examination and a routine biochemistry workup.

A clean procedure was defined as an elective surgery, with or without the use of prosthetic devices, in which no inflammation was encountered, and during which the respiratory, alimentary and genitourinary tracts were not entered [11].

Antibiotic prophylaxis was chosen based on international guideline recommendations [9,11,12] for the type of surgery. Consistent with the guidelines for clean procedures, cefazoline was used as a standard weight-based dosage and, in allergy sufferers, clindamycin were the main antibiotics used for surgical prophylaxis. The administration of the first dose of antimicrobial was given within 60 min before surgical incision. The choice of whether or not to use prophylaxis depended on the indications of the guidelines [9] based not only on the type of intervention, but also on the positioning of the prosthesis. All surgical procedures followed standardized surgical techniques and were performed by the same team of surgeons, which consisted of a senior surgeon and one or more doctors in training. Moreover, the same surgical intra-operative and monitoring procedures were used in all enrolled cases.

**Figure 1:**
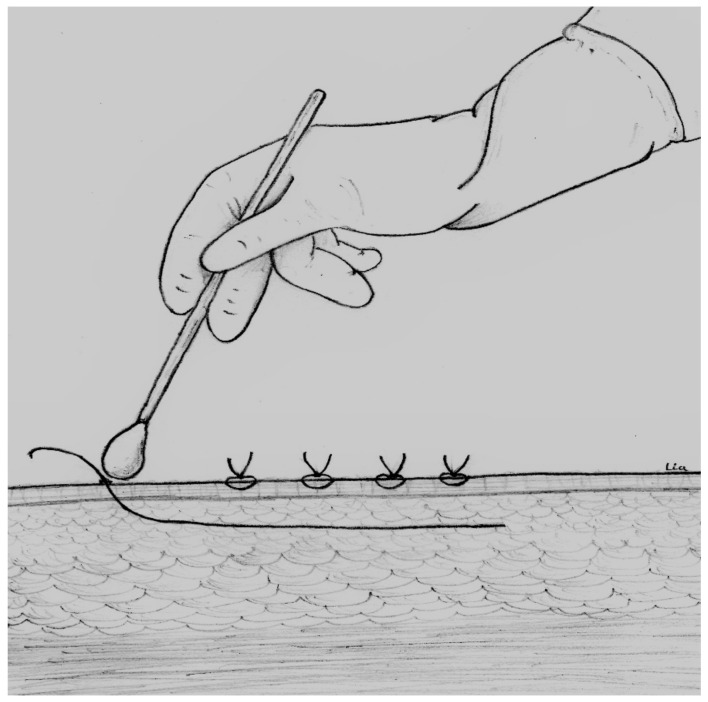
Wound swab at the cutaneous exit point of the suture thread.

A gauge 2 synthetic absorbable suture thread (Coated, Braided Lactomer 9–1) between 5 and 10 cm long [13,14], normally used in clinical practice, was inserted into the surgical sites of all patients at the end of surgery. After 48 h, the skin was properly disinfected with chlorhexidine 2%, in order to reduce contamination; the internal part of the thread, which remained under the skin, was removed and sent to the laboratory for microbiological analysis. In addition, a wound swab was made at the cutaneous exit point of the suture thread (Figure 1). The swab was sent for a microbiological examination, to evaluate the possible contamination of the extracted thread by the bacteria present on the skin at the cutaneous exit point. A thread was considered colonized when the presence of bacteria, without apparent clinical symptoms or tissue reaction, was observed by microbiological methods.

In the laboratory, the suture threads were centrifuged for 30 s and exposed to low frequency (40 kHz) ultrasound for 60 s [15]. All microbiological analysis was carried out using an ultrasonic bath for the sonication procedure. After sonication, a new centrifugation was carried out. The sediments were cultured on aerobic agar plates and incubated in an aerobic atmosphere at 37 °C. Microbiological identification was made using the automated bacterial identification system Vitek2 (bioMerieux). Antimicrobial susceptibility testing was performed using the EUCAST disk diffusion method and Vitek2. For more detailed information on microbiological methods, refer to Iovino et al. [10].

Post-operative wound management was carried out by a specialized tissue viability nurse and junior doctors using an aseptic, non-touch technique for changing or removing dressings and, if necessary, by removing the devitalized tissue or exudates, which may otherwise have delayed wound healing. In all cases, sterile gauze dressing and chlorhexidine 2% for wound cleaning were utilized. Patients were followed up for 28 days in the absence of prosthesis, and for 90 days in the presence of it. Surgical site infection (SSI) was defined on the basis of the combined clinical judgment of the infectious disease expert and senior surgeon. A skin swab was performed on each patient with suspected SSI; while awaiting the results of the culture, a large spectrum antibiotic therapy was started. For all patients with an SSI, basic demographics, infection characteristics and the outcome at hospital discharge were collected.

### 2.3. Statistical Analysis

Means and standard deviations were used to summarize the continuous variables, whereas absolute and relative frequencies were used to indicate the categorical variables.

A Student *t*-test was used to evaluate the differences for the continuous variables, whereas a chi-square test was used for the categorical variables, using exact procedures if necessary.

## 3. Results

A total of 238 patients were enrolled: 208 (87.4%) were subjected to clean procedures without the placement of prosthesis, and 30 (12.6%) with the prosthesis. The clinical background and the demographic features of the enrolled patients are shown in Table 1. The average duration of the surgical procedure was 50–75° quartile. Of the 238 patients enrolled, 117 (49.2%) underwent an antimicrobial prophylaxis. All patients subjected to clean surgical operations with prosthesis placement received antibiotic prophylaxis; among the patients who underwent a clean procedure without the placement of prosthesis, only 87 (41.8%) received antibiotic prophylaxis because indicated.

An total of 79 (33.2%) patients showed a bacterial colonization of the thread with bacteria different to that observed at the point of the insertion of the suture thread. The characteristics of the patients according to the presence of thread colonization are shown in Table 2.

All the skin swabs performed were negative. Regarding the 208 patients without the implantation of prosthesis, a colonized thread was observed in 19 (21.8%) of the 87 with antibiotic prophylaxis, and in 58 (47.9%) of the 121 without it. Among the 30 patients with the implantation of prosthesis, only two patients showed a colonized thread. Thus, the patients with antibiotic prophylaxis developed a colonization of the thread less frequently than those patients without it (17.9% vs. 47.9%, *p* < 0.001).

**Figure 2:**
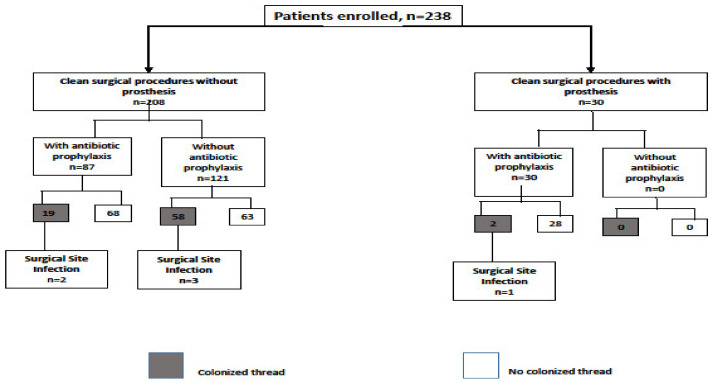
Flow chart of study participant recruitment.

The relationship between antibiotic prophylaxis, the bacterial colonization of the suture thread and SSIs are shown in Figure 2. An SSI was observed in six (2.5%) patients; all SSIs were superficial. No difference in the rates of SSIs was observed between the patients with antibiotic prophylaxis and those without it (2.6% vs. 2.5%, *p* = 0.96), while a correlation between colonized thread and SSI was observed. Precisely, SSI was observed in 5 (6.4%) of 77 patients without prosthesis and a colonized thread, in one (50%) of the two patients with the implantation of prosthesis and a colonized tread, and in none of the 159 (with or without prosthesis), but without a colonized thread. Thus, SSI was detected more frequently in patients with a colonized thread after surgery procedure (7.6% vs. 0%, *p* < 0.001). Among the 174 cancer patients, most of them were early breast cancer or melanoma stage I – II, and none were receiving chemotherapy or immunotherapy at the time of the surgical procedure. Moreover, there was no statistically significant difference in the prevalence of suture contamination or SSIs between cancer patients compared to non-cancer patients (35.6% vs. 26.5%, *p* = 0.15; 2.9% vs. 1.6%, *p* = 1, respectively). Also, no statistically significant difference in BMI was observed between patients with suture thread contamination compared to a group without (mean ± SD: 25.82 ± 1.35 vs. 26.09 ± 1.32 *p* = 0.15), and between those with or without SSI (26.33 ± 1.33 vs. 25.67 ± 1.35 *p* = 0.25).

The main demographic and clinical characteristics of the patients with SSIs are shown in Table 3. Interestingly, the microorganisms identified in the colonized thread were the same as those found in SSIs, and were mainly Gram-positive cocci.

## 4. Discussion

Despite improvements in their prevention, SSIs remain a significant clinical problem as they are associated with substantial mortality and morbidity. The incidence of SSIs may be as high as 20%, depending on the surgical procedure, the surveillance criteria used, and the quality of data collection [16].

The incidence of SSIs was low (less than 4%) in clean surgical procedures, so the indication of antibiotic prophylaxis was limited only in some procedures. However, the management of the risk of SSIs in clean surgical procedures is still under debate. Thus, the identification of a method that favours the identification of the patients at risk of SSIs after clean surgical procedures, and of the bacteria involved, seems to be relevant in the clinical practice.

In the present study, we evaluated the role of the colonization of a suture thread in 238 patients who underwent a clean surgical procedure in identifying the patients who may develop a surgical site infection and the bacteria involved. In our analysis, we found an SSI rate of 2.5%, comparable to that described in the literature for clean surgical procedures. A colonized thread was observed in 79 (33.2%) patients; more specifically, in 77 (37%) patients subjected to clean procedures, without the placement of prosthesis, and in two (6.6%) patients with prosthesis.

Despite some studies having reported a significant correlation between existing cancer and the likelihood of SSIs [7,17], this relationship was not found in our study.

Surgical suture can be contaminated by bacteria from the environment or from the patient’s normal flora [18]. There are no definitive data on the incidence of SSIs being directly associated with sutures, but it is reasonable to assume that a substantial proportion of SSIs involves sites containing suture materials, and that the suture thread itself provides a nidus for microbial adherence and wound contamination. Therefore, it could be a factor leading to the colonization/infection mechanism.

Antibiotic prophylaxis is effective in reducing thread colonization at the end of surgery, bacterial adhesion, and the probable occurrence of infections.

SSI was registered in six patients, one with prosthesis and five without it. However, only patients who showed colonization of the suture thread then developed a surgical site infection. In fact, no patient without colonization of the thread developed SSI.

Therefore, this method may be useful in the management of surgical patients, since it may be an indicator of the potential risk of the development of a surgical site infection.

However, many studies have shown that properly administered prophylactic antibiotics could prevent postoperative infection [19,20], and we found no difference in the rate of SSIs between patients with antibiotic prophylaxis and patients without it.

Moreover, we found a relationship between the bacteria isolated in the thread and the one responsible for SSI. This finding can be advantageously used by the clinician to set up a therapy which is as targeted as possible, using germs isolated from the thread, thus reducing the use of the broad-spectrum antibiotic treatments that are increasingly responsible for the worldwide phenomenon of antimicrobial resistance. In fact, the use of targeted and narrow-spectrum antibiotic therapies has a relevant significance in the antimicrobial stewardship programs, reducing the selective pressure on hospital and community bacteria, and thus the development of multi-drug resistant microorganisms [21,22,23,24].

In conclusion, in the clean surgical procedures we found a significant association between the presence of colonized thread and SSI risk. Moreover, the bacteria identified on the thread at 48 h after the procedure were those responsible for SSI later, allowing clinicians to start a specific antimicrobial therapy; thus, it seems to be relevant in the clinical practice. Further clinical investigations are warranted in larger studies to confirm these results, which should also use a control group with sutures that are already covered with antimicrobial film.

## Figures and Tables

**Table 1:** Clinical background and demographic features of enrolled patients.

N° of Patients	238
Age, median (range)	58 (19–88)
Males, n (%)	112 (47.1)
Type of procedure, n (%)	
- Clean without prosthesis - Clean with prosthesis	208 (87.4) 30 (12.6)
ASA score, median (range)	2 (1–3)
BMI, median (range)	26 (24–29)
Patients with antibiotic prophylaxis, n (%)	117 (49.2)
Patients with cancer, n (%)	174 (73.1)

**Table 2:** Characteristics of the patients enrolled, according to the presence of thread colonization.

Characteristics	Thread Colonization N = 79 (33.2%)	No Thread Colonization N = 159 (66.8%)
Age median, (range)	57 (21–83)	58 (19–88)
Males, n (%)	31 (39.2)	81 (50.9)
Type of procedure, n (%)		
Clean without prosthesis Clean with prosthesis	77 (97.5) 2 (2.5)	131 (82.4) 28 (17.6)
ASA, median	2	2
BMI, median (range)	26 (24–28)	25 (24–29)
Patients with antibiotic prophylaxis, n (%)	21 (26.6)	96 (60.4)
Patients with cancer, n (%)	62 (78.5)	112 (70.4)
SSI, n (%)	6 (7.6)	0

**Table 3:** Characteristics of patients with a surgical site infection (SSI), and correlation between bacteria isolated on the colonized thread and bacteria responsible for SSI.

Patients with SSI	N1	N2	N3	N4	N5	N6
Age	71	57	72	58	63	45
Sex	F	F	M	M	M	M
Operation	Clean (quadrantectomy with lymph node dissection)	Clean (mastectomy with lymph node dissection)	Clean with prosthesis (inguinal hernia repair)	Clean (cutaneous melanoma radicalization)	Clean (sentinel lymph node biopsy)	Clean (sentinel lymph node biopsy)
Prosthesis	No	No	Yes	No	No	No
Cancer	Yes	Yes	No	Yes	Yes	Yes
ASA	2	2	1	2	3	2
Antibiotic prophylaxis	No	No	Yes	Yes	Yes	No
Duration of surgery	75 *	<75 *	50 *	50 *	<75 *	<75 *
Antibiotic therapy after procedure	No	No	No	No	No	No
Skin swab	Negative	Negative	Negative	Negative	Negative	Negative
Bacteria identified on the colonized thread	*Staphylococcus epidermidis/* *Enterococcus faecalis*	*Kocuria rosea/* *Staphylococcus hominis*	*Enterococcus faecalis*	*Staphylococcus aureus*	*Staphylococcus aureus*	*Staphylococcus lugdunensis*
Bacteria responsible for SSI	*Enterococcus faecalis*	*Kocuria rosea*	*Enterococcus faecalis*	*Staphylococcus aureus*	*Staphylococcus aureus*	*Staphylococcus lugdunensis*

* Percentile of the surgical duration.
